# Patients’ Experiences and Priorities for Accessing Gastroenterology Care

**DOI:** 10.1093/jcag/gwz029

**Published:** 2019-10-30

**Authors:** Jennifer J Telford, Gregory Rosenfeld, Swati Thakkar, Nick Bansback

**Affiliations:** 1 St. Paul’s Hospital, Division of Gastroenterology, Department of Medicine, University of British Columbia, Vancouver, British Columbia, Canada; 2 Consultant; 3 Department of Medicine, School of Population and Public Health, University of British Columbia, Vancouver, British Columbia, Canada

**Keywords:** Discrete choice experiment, Patient preferences, Wait time

## Abstract

**Background:**

Wait times for gastroenterology care in Canada exceed recommended benchmarks set by the Canadian Association of Gastroenterology wait-time consensus. Patient-centered prioritization tools may help improve efficiency.

**Methods:**

We conducted a survey on gastroenterology outpatients assessing their experience with accessing care, global health status and health care service utilization while waiting for a gastroenterology appointment. Thematic analysis of survey results informed the questions for a discrete choice experiment (DCE). Three attributes included were the following: clinical indication, functional status and time already waiting, which the study patients considered when prioritizing hypothetical patients. The DCE was analyzed using a conditional logit model.

**Results:**

One hundred seventy-three patients completed all questions and were included in the final analysis. Over 80% reported good or excellent physical and mental health with 11% utilizing health care resources while waiting; 14% had waited more than 25 weeks for their appointment. Seventy-seven per cent of the patients were satisfied or better with their experience. Eighty-one per cent of the patients agreed with a prioritization system. Patients would prioritize a patient with a potentially more severe diagnosis or functional impairment over a patient with a less severe diagnosis clinical or functional impairment who had been waiting longer. The most severe clinical attributes were prioritized over the most severe functional attributes.

**Conclusion:**

Patients support a prioritization tool for access to gastroenterology care. DCE indicated that patients are willing to wait longer in order for those with more severe clinical or functional attributes to be seen earlier. The relative times patients are willing to wait could be used to create a prioritization model for outpatients referred to gastroenterology.

## Background

In many jurisdictions, access to gastroenterology care is limited by a lack of gastroenterologists and endoscopy resources to serve the population. According to the most recent Canadian Association of Gastroenterology (CAG) survey, the average wait time for gastroenterology care is approaching 160 days and wait times for urgent indications such as active inflammatory bowel disease were an average of 66 days after referral (1). The CAG consensus guidelines on appropriate wait times for gastroenterology care recommends these patients be seen within 14 days (2). In an unpublished quality audit of gastroenterology patients undergoing endoscopic procedures at St. Paul’s Hospital from January to March 2014, only 7% (13/185) of our patients met CAG wait-time guidelines for urgent or semi-urgent indications.

The predominant approach to wait list management is first-in/first-out, often with an ad hoc prioritization for urgent cases (3,4). The limitations of such an approach is that more serious cases may not be seen in time to prevent irreversible harm, or patients with milder symptoms may never reach the top of the list (if there is a steady influx of more urgent patients). Furthermore, when urgent cases are managed by creating open blocks each week, waste can occur if these appointments are not filled.

In many clinical areas, there is a move towards prioritization tools to manage waiting lists and improve efficiency. These tools tend to classify patients with a summary score based on their clinical attributes, with patients with higher scores given priority. The advantage of these tools is that they make explicit the criteria for prioritization improving the fairness and health outcomes to patients (5,6). While public and clinician input has generally been supportive of the idea of explicit prioritization (3,7), the method used to assign prioritization scores is controversial. Focusing solely on clinical attributes may miss nonclinical aspects that are considered important to patients. Moreover, there is a wealth of evidence suggesting that there are important differences between the way doctors prioritize by using clinical and nonclinical attributes in comparison to the way patients would prioritize (8). Failing to consider what matters to patients may lead to dissatisfaction or a sense of unfairness with the prioritization process (6).

The objective of this study was to further understand patients’ experiences with gastroenterology care, and determine priorities for how the wait list should be redesigned. Specifically, we aimed to determine the resources patients waiting for gastroenterology care had used, how waiting has impacted their quality of life, and how patients would prioritize future patients based on their clinical diagnoses, functional impairment and waiting time.

## Methods

We conducted a survey in patients at an outpatient, general gastroenterology clinic. The referred patients were either new to the clinic or returning patients whom had not been seen in the preceding 6 months. The survey consisted of questions on patients’ experience on accessing gastroenterology care, their global health status and health care service utilization while waiting for a gastroenterology appointment, and discrete choice experiment (DCE) questions to understand how patients would prioritize hypothetical patients with different diagnoses and functional outcomes. DCEs were developed in marketing research (9) but have become increasingly popular in health services research and have been used to explore a range of health-related services and treatments (10). DCEs work on the premise that any product or service can be described by levels of its characteristics, known as attributes, and that the extent to which an individual values the product or service is dependent on a weighted sum of the levels of these characteristics (11). DCEs are underpinned by random utility theory (12), which states that the probability that product A is chosen over product B is proportional to how much product A is valued over product B. Ethical approval was granted from the University of British Columbia behavioural ethics board.

### Survey Development

To develop the survey questions, we interviewed 20 patients about their experiences of care, and their perspectives on the attributes that should guide prioritization for different patients. We recruited patients who had been seen in consultation during October 2015 and obtained their telephone permission to be contacted regarding their care experience.

To derive attributes for use in the DCE, we used a thematic analysis (13). This analysis suggested three key attributes should be included: clinical indication, functional ability, and time already waiting. To define levels for these attributes, we used the Canadian consensus on medically acceptable wait times for digestive health care (2). For the indication, which varied from ‘no gastrointestinal symptoms but is at high risk of cancer and needs a preventative test’ to ‘patient has symptoms that may include pain, blood in the stool or a new change in the bowel movements, the patient requires evaluation by a gastroenterologist to find the reason for the symptoms and start treatment’. For function, we used a modified version of the Eastern Cooperative Oncology Group scale of performance which varied from ‘the patient is fully active, able to carry on all daily activities without restrictions’ to ‘the patient is capable of only limited self-care; confined to bed or chair more than 50% of waking hours’. We made sure it was clear that functional impairment was due to gastrointestinal symptoms. For wait times, we used levels varying from 1 week to 24 weeks, which reflected wait times in the clinic (Table 1).

**Table 1.** Attributes and levels in the discrete choice experiment

Attribute	Level #	Level description
**Diagnosis**	1	Patient has long-standing symptoms that can be managed by a family doctor but the patient would like to see a gastroenterologist for help with residual symptoms.
	2	Patient has no gastrointestinal symptoms but requires an elective screening test for cancer.
	3	The patient has an abnormal test which may indicate a gastrointestinal disease but needs a procedure to confirm diagnosis.
	4	Patient has long-standing diarrhea. The patient requires evaluation by a gastroenterologist to find the reason for the symptoms and start treatment.
	5	Patient has no gastrointestinal symptoms but is at high risk of cancer and needs a preventative test.
	6	Patient has symptoms that may include pain, blood in the stool or a new change in the bowel movements. The patient requires evaluation by a gastroenterologist to find the reason for the symptoms and start treatment
**Function**	1	The patient is fully active, able to carry on all daily activities without restrictions.
	2	The patient is restricted in physically strenuous activity but ambulatory and able to carry out work of a light or sedentary nature, e.g., light house work, office work
	3	The patient is ambulatory and capable of all self-care but unable to carry out any work activities; up and about more than 50% of waking hours
	4	The patient is capable of only limited self-care; confined to bed or chair more than 50% of waking hours.
**Time waiting**	1	24 weeks
	2	12 weeks
	3	4 weeks
	4	1 week

The survey began with questions about the socio-demographics and quality of life of the participants. We also asked questions about participants’ experiences of care and their utilization of services while they were waiting for care.

### Experimental Design and Construction of Choice Sets

**Figure 1.**
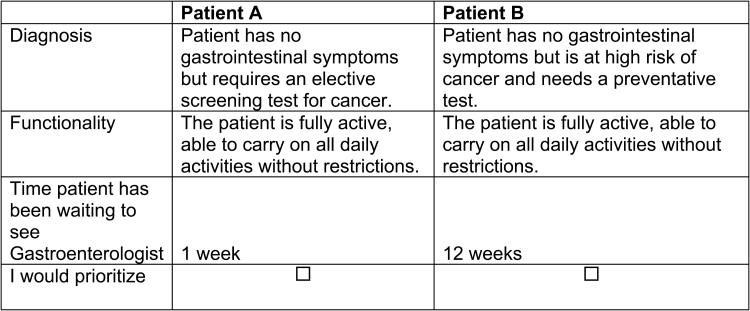
Example choice set. Which patient would you prioritize?

Including three attributes, with 6, 4 and 4 levels, respectively, gives a full factorial of 96 possible combinations of levels, which means there could be 9120 possible pairwise choice sets. To provide a manageable task for respondents, we used the D-optimality criterion to maximize the efficiency of the design. Forty choice sets with two alternatives comprising of different sets of attribute levels were constructed using NGene Software (ChoiceMetrics, Sydney). To make the questionnaire more feasible, we randomly blocked the 40 choice sets into 4 sets of 10 choices. Consequently, each respondent was provided with 10 DCE choice sets presenting a scenario where they had to choose between prioritizing two hypothetical patients with different type of diagnosis, level of function and time waiting for care (Figure 1).

We estimated that 50 responses for each choice set would provide sufficient numbers for evaluating heterogeneity in preferences between respondents. Assuming a 50% response rate, we sought to recruit 200 individuals.

### Analysis

We analyzed the DCE responses using the conditional logit model (12). Briefly, this assumes that an individual’s utility function can be defined by the levels of each attribute and the life years in each scenario. We used effects coding for the indication for referral and functional attribute levels, and after testing levels, assumed wait time to be a linear function.

$$\begin{array}{ll} U=\; & \alpha \;+\;\beta_{1}Diagnosis_{1}\;+\;...\;+\;\beta_{6}Diagnosis_{3}\;+\;\beta_{7}Function_{1}\\ & \;+\;....\;+\;\beta_{10}Function_{3}\;+\;\beta_{11}Wait\;time\;\varepsilon \; \end{array}$$

The marginal willingness to wait, calculated as each attribute level coefficient divided by the wait time coefficient, indicates the relative importance of each attribute level compared to each other. We use the values to interpret weights for prioritizing future patients.

## Results

Of the 198 patients who consented to participate in the survey, 173 completed all questions and were included in the final analysis. Patients were well educated (98% of patients had high school or higher education) and 95% of the patients had a family physician (Table 2).

**Table 2.** Demographics

Year of birth	*N*	%
1940–1949	35	20%
**1950–19**59	58	34%
**1960–19**69	41	24%
**1970–19**79	21	12%
**1980**+	18	10%
**Gender**		
**Male**	81	47%
**Female**	92	53%
**Highest Level of Education:**		
**8th grade or less**	2	1%
**Some high school**	3	2%
**High school**	36	21%
**College**	52	30%
**Undergraduate**	36	21%
**Postgraduate**	44	25%
**Have a family Physician**		
**Yes**	165	95%
**No**	8	5%
**Frequency of visit to family Physician for Gastroenterology care**		
**None**	15	9%
**Once a year**	88	51%
**Twice a year**	22	13%
**More than two times a year**	48	28%
**In general, how would you rate your overall physical health?**		
**Excellent**	31	18%
**Good**	104	60%
**Fair**	27	16%
**Poor**	9	5%
**Very poor**	2	1%
**In general, how would you rate your overall mental health?**		
**Excellent**	53	31%
**Good**	92	53%
**Fair**	22	13%
**Poor**	4	2%
**Very poor**	2	1%

Eighty per cent of the patients rated having good or excellent physical health and 85% rated having good or excellent mental health. Given the positive patient perception of their physical health, it was not surprising to find the majority of the patients did not use any other services while waiting to see the specialist. 11% of respondents used home care services or walk-in clinics while waiting to see a specialist and 14% used emergency services (Table 3). Fourteen per cent of the respondents had waited 25 weeks or more to see their gastroenterologist for the first time.

**Table 3.** Services used while waiting to see the specialists for your gastro complaint after referral

Emergency Services/Hospital		
**None**	145	84%
**1–3**	22	13%
**4–6**	5	3%
**7–9**	1	1%
**10 and up**	0	0%
**Family Physician**		
**None**	89	51%
**1–3**	62	36%
**4–6**	17	10%
**7–9**	1	1%
**10 and up**	4	2%
**Home care services**		
**None**	151	87%
**1–3**	15	9%
**4–6**	6	3%
**7–9**	0	0%
**10 and up**	0	0%
**Walk-in clinics**		
**None**	153	88%
**1–3**	14	8%
**4–6**	6	3%
**7–9**	0	0%
**10 and up**	0	0%
**How long did you wait to see the Gastroenterologist for the first time?**		
**1 week or less**	9	5%
**2–4 weeks**	42	24%
**5–12 weeks**	70	40%
**13–24 weeks**	28	16%
**25 weeks or more**	24	14%

### Patient Experiences

The majority of the patients were satisfied with their gastroenterology care (Table 4). Seventy-seven per cent of the respondents were satisfied or better (very satisfied, extremely satisfied) with the time that they waited to see the specialist after first referral. Similarly, 82% were satisfied with the ease of making a follow-up appointment and 82% were also satisfied with the ability to access a gastroenterologist through the family physician. Overall, the vast majority of the patients (90%) were satisfied with the care of the gastroenterologist.

**Table 4.** Results of experiences

	Extremely dissatisfied	Very dissatisfied	Satisfied	Very satisfied	Extremely satisfied	N/A
**Ability to access the Specialist through your Family Physician?**	6 (3%)	18 (10%)	57 (33%)	59 (34%)	43 (25%)	0 (0%)
**The wait time to see the Specialist first time after making a referral?**	16 (9%)	24 (14%)	72 (42%)	37 (21%)	24 (14%)	0 (0%)
**Ease of making follow-up appointments with the Specialist?**	9 (5%)	16 (9%)	69 (40%)	44 (25%)	29 (17%)	6 (3%)
**Overall Gastroenterologist care at the office?**	8 (5%)	7 (4%)	49 (28%)	59 (34%)	48 (28%)	2 (1%)

### Patient Preferences

Overall, 81% of the patient respondents agreed that a prioritization system would be a good solution. In the DCE questions, all respondents chose at least once to prioritize a patient with either a more severe indication for referral or functional level at the cost of a less severe patient waiting longer.

As expected, the wait-time coefficient was negative indicating patients preferred to wait less time. The priority of patients in terms of their function and indication increased by increasing level of severity for both (Table 5). The worst three indication levels were considered more of a priority over the worst functional level.

**Table 5.** Conditional logit results

Attribute	Coef.	Std. Err.	*P*	95% CI	Willingness to wait (weeks)
**Diagnosis**	-				
** Level 1**	ref	-	-	-	59
** Level 2**	0.52	0.23	0.024	(-0.07–0.98)	49
** Level 3**	1.43	0.20	<0.001	(1.01–1.86)	30
** Level 4**	1.80	0.21	<0.001	(1.38–2.21)	23
** Level 5**	2.44	0.21	<0.001	(2.04–2.85)	10
** Level 6**	2.95	0.22	<0.001	(2.54–3.36)	0
**Function**					
** Level 1**	Ref	-	-	-	35
** Level 2**	1.00	0.13	<0.001	(0.74–1.26)	15
** Level 3**	1.21	0.15	<0.001	(0.92–1.50)	11
** Level 4**	1.74	0.14	<0.001	(1.46–2.02)	0
**Wait Time (weeks)**	−0.05	0.00	<0.001	(0.04–0.06)	-

Conditional (fixed-effects) logistic regression Number of obs = 3114.

LR chi^2^ (9) = 872.32

Prob > chi^2^ = 0.0000

Log likelihood = −1430.8199 Pseudo R^2^ = 0.2336.

Within the indication attribute, in comparison to a patient with ‘symptoms that may include pain, blood in the stool or a new change in the bowel movements’ (level 6), a patient with ‘long-standing symptoms that can be managed by a family doctor but the patient would like to see a gastroenterologist for help with residual symptoms’ (level 1) should be willing to wait an additional 59 weeks, and a patient with ‘no gastrointestinal symptoms but requires an elective screening test for cancer’ (level 2) should be willing to wait 49 weeks. Levels 3, 4 and 5 had estimated willingness to waits of 30, 23 and 10 weeks, respectively. It is important to note, these results do not suggest a patient would be content to wait this long—rather, that within the current wait times, the weeks provide an illustration of how much less important each clinical indication for referral is when compared to the most severe level.

In terms of function, in comparison to a patient who is ‘is capable of only limited self-care; confined to bed or chair more than 50% of waking hours’. (level 4), a patient who is ‘fully active, able to carry on all daily activities without restrictions’ (level 1) should be willing to wait an extra 35 weeks, and a patient who ‘is restricted in physically strenuous activity but ambulatory and able to carry out work of a light or sedentary nature, e.g., light house work, office work’ (level 2) should be willing to wait 15 additional weeks.

## Discussion

This project sought to provide preliminary information to demonstrate how a patient-centred prioritization tool for patients seeking gastroenterology care could be derived. Such a tool would reflect third-party payer priorities for health care becoming more patient centred. Our sample included many patients who had waited a considerable time for gastroenterology care, and over 1 in 10 had received emergency care while waiting. But overall, the majority of patients were satisfied with their experiences. Our main findings are first that patients are generally willing to prioritize wait times based other patients level of severity, and second, that severity should be based on both clinical characteristics and functional abilities. We found that severe clinical characteristics seem more important than severe functional characteristics.

The results of this study could be used to develop a tool that determines the wait list for patients. Referring physicians could indicate the clinical severity, waiting time, and other factors that patients deem relevant (such as the impact of their condition on work and family). A patient with the same clinical indication as another, but with worse functional status, would be scheduled earlier. Each day, the tool would produce an updated prioritization score for each patient, which will be used to prioritize patients to available appointments with GI specialists. Given that the score will weigh the number of weeks on the wait list, milder patients will be seen in a systematic way despite the perpetual influx of patients meeting more severe clinical and/or functional criteria. The advantage of this approach is that it is based on patients’ priorities, and considers more than just clinical attributes. The hypothesis is that when patients are involved in this process, waiting becomes more understandable and transparent to patients. This also enables patients to be seen in the most efficient manner.

Since this study sought to provide a proof of concept, there are some important limitations that must be considered when extrapolating the results to actual practice. Most importantly, our estimates of willingness to wait should not be interpreted literally. They are based on the levels we used in the study, which reflected the current wait times in the local clinic. Instead, they should be interpreted as the relative time that patients could wait, and reflect the current demand and capacity in the local clinic. They could be recalculated based on a different reference point if capacity was increased. Second, while respondents were told the responses were anonymous, since surveys were filled in the waiting room, and patients might have been concerned about reporting their honest experiences of care. For example, a majority of respondents were satisfied with the current wait time to see the specialist. Third, the patients were highly educated, had a regular family doctor and many of the patient’s surveyed were followed regularly by a gastroenterologist. Response bias may have influenced the representativeness of individuals who chose to participate and so our sample. The influence of these patient factors on the prioritization of attributes is unknown. Fourth, the generalizability of results should consider that this study was conducted in a universal health care setting where patients are not paying for care. Fifth, functional states were derived from the Eastern Cooperative Oncology Group scale, which was developed for the assessment of patients with cancer rather than gastrointestinal disease. Finally, our experimental design for the DCE did not consider the interactions between function and clinical presentation, which could have important implications for deriving a wait time model.

Wait time for specialist care, including gastroenterology, is an issue in Canada as well as other countries with publically funded health care systems (1,14,15). A survey of gastroenterology patients from five Canadian provinces indicated that gastroenterology symptoms accounted for 23% missing work or school in the preceding month, 18% reporting significant interference with social functioning and 15% significant interference with activities of daily living (14). Respondents felt no patient should wait more than 3 months to be seen by a gastroenterologist. In contrast to asking patients a maximum wait time, a strength of our study is seeking relative wait times for different levels of clinical and functional severity, considering the limited capacity and resources.

In a related study, Moayyedi et al. used a DCE to assess patient preferences regarding four different aspects of outpatient evaluation in the United Kingdom (15). They found that patients valued the wait to see a gastroenterologist in consultation equally to the wait for a diagnostic test following their consultation. Patients were willing to wait longer to see a specialist in consultation if they did not have to wait as long for investigations. Preferred scenarios were a longer wait time to consultation with a subsequent short wait time to testing or, ideally, a consult and diagnostic testing (i.e., endoscopy) at the same time. The present study was focussed more on the prioritization of patients, not on the type of care patients received. We envisage future studies that consider both prioritization and type of care attributes together. In these future studies, we propose that effort is made to recruit patients that are more representative of the whole GI population, and envisage that simplified questions are used routinely so that a prioritization algorithm can learn over time, and also act as a way to hear better the concerns of patients (16).

In conclusion, examination of gastroenterology preferences regarding wait-time prioritization using a DCE indicated that patients supported others with more serious clinical symptoms or functional status having a shorter wait time. This knowledge may be helpful to develop a patient-centered waiting list prioritization tool. Future studies evaluating patient perceptions of the relationship between clinical presentation and different models of delivery of care are needed to further enhance the development of appropriate wait time prioritization tools. Futures studies are needed to evaluate patient perception of the relationship between clinical presentation and different models of care, such as proceeding directly to endoscopic evaluation.
